# Case Report: A case of complex cortical dysplasia with central precocious puberty onset

**DOI:** 10.3389/fped.2025.1634704

**Published:** 2025-08-29

**Authors:** Chong Guo, Chunmei Chen, Xinrui Wang, Kaiwu Lin, Jingmin Guo, Pin Ge

**Affiliations:** ^1^ Child Healthcare Department, Fujian Maternity and Child Health Hospital College of Clinical Medicine for Obstetrics & Gynecology and Pediatrics, Fujian Medical University, Fuzhou, China; ^2^ Medical Genetic Diagnosis and Therapy Center, Fujian Maternity and Child Health Hospital College of Clinical Medicine for Obstetrics & Gynecology and Pediatrics, Fujian Medical University, Fuzhou, China

**Keywords:** case report, complex cortical dysplasia, central precocious pubertybeta, tubulinopathy, TUBB2B

## Abstract

A mutation rendering microtubulin and microtubule-associated proteins ineffective leads to a tubulinopathy known as complex cortical dysplasia (CCD), characterized by clinical heterogeneity and a variety of cortical brain developmental abnormalities. These often commence with intellectual disability, epileptic seizures, and motor disorders. In this case, we present a 6-year-old child with CCD whose symptoms began with central precocious puberty. Whole-exome sequencing uncovered a TUBB2B gene heterozygous mutation, NM_178012.5:c.74G > A (p.Ser25Asn). To our knowledge, this mutation has not been previously documented. Computational structural analysis indicated that this variant alters the hydrogen bonding between Ser25 and Trp21, Gly29, and Ile30, thus modifying the secondary structure and function of the protein, contributing to the child's unique clinical presentation. These findings expand the range of TUBB2B gene variants and offer a direction for the precise treatment of this child, underscoring the importance of brain magnetic resonance imaging in children with central precocious puberty.

## 1 Introduction

Complex cortical dysplasia (CCD) is an autosomal dominant condition, characterized by a clinical heterogeneity and a diverse range of cortical brain developmental abnormalities. The most profoundly impacted individuals, often fetal, exhibit microlissencephaly, cortical plate absence, agenesis of the corpus callosum, along with severely underdeveloped brainstem and cerebellum. Additional cases may present with lissencephaly, polymicrogyria, cortical dysplasia, or neuronal heterotopia. Patients with milder malformations may survive, typically experiencing varying levels of neurological impairment including mental retardation, seizures, and movement disorders (1, 2).

This case involves a girl diagnosed with CCD at the age of 6 years and 5 months, and it is characterized by the following key features: Firstly, her initial symptom was central precocious puberty, and she exhibited no classic signs of complex cortical developmental abnormalities either during the fetal period or after birth. Secondly, genetic analysis revealed that she harbors a *de novo* mutation in the TUBB2B gene, which to our knowledge, has not been previously reported.

## 2 Case description

**Figure 1:**
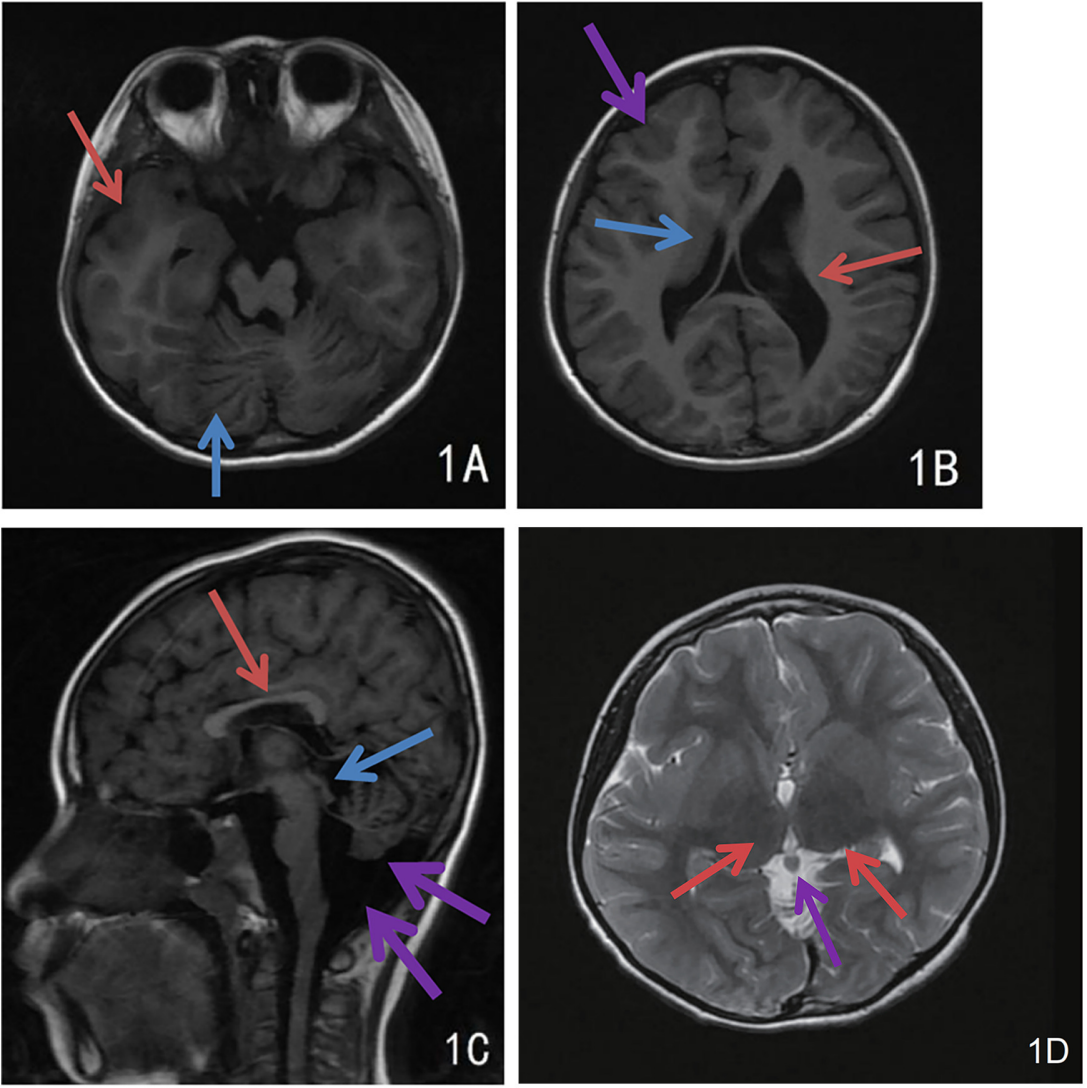
Cranial MRI findings of the child. **(A)** Extensive and deep cerebellar sulcus in the cerebellar hemispheres (blue arrow), cortical thickening and dysplasia of the right temporal lobe (red arrow); **(B)** Uneven cortical hypertrophy, midline deviation, dilated lateral ventricles and hypoplasia of the right frontal lobe, irregular cortical morphology of right frontal lobe with dysplasia (purple arrow), hypertrophic caudate nucleus at right ventricular border (blue arrow), dilatation of left ventricle (red arrow); **(C)** Irregular cortical hypertrophy, short corpus callosum (red arrow), hypoplasia of the cerebellar earthworms with widened retrocerebellar CSF space (purple arrow), and heterogeneous signal intensity in pineal region (blue arrow); **(D)** Bilateral thalamic hypertrophy (red arrows), nodular shadow in the pineal region (purple arrow), possibly a pineal cyst.

**Figure 2:**
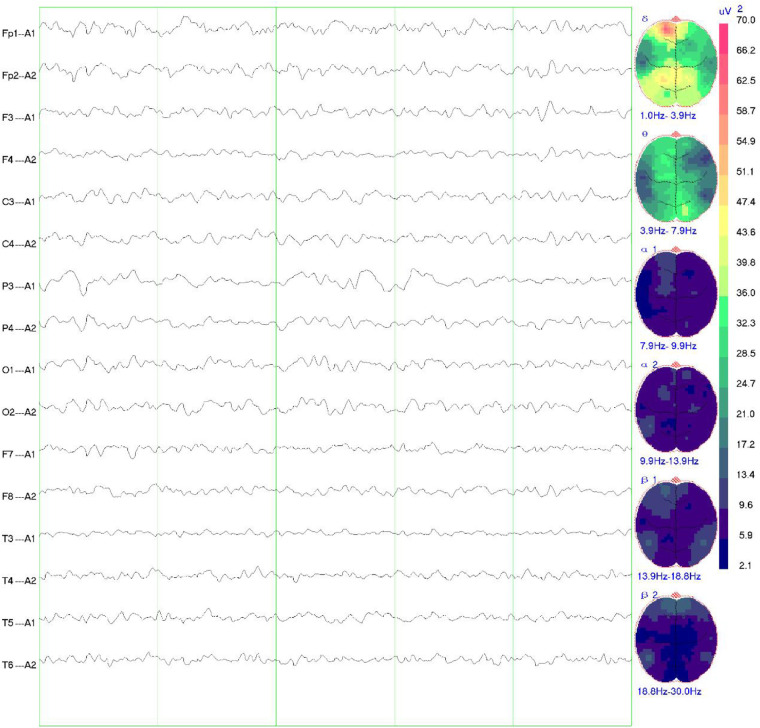
EEG findings in the child. The video EEG showed no dominant rhythm in the occipital region in the awake, quiet and closed-eye state, 6–8 Hz low-amplitude mixed activity interspersed with a few low-amplitude fast waves, roughly symmetrical to the left and right, and no bilateral epileptiform discharges.

Girl child, 6 years and 5 months old, presented with bilateral breast development for one week. The mother's prenatal history was unremarkable. The language domain developed normally from an early age, but the gross motor domain showed delayed development. The child could produce intentional vocalizations at 12 months of age, but did not achieve independent walking until 16 months. There is currently significant motor coordination impairment, including an inability to stand on one foot, walk on a balance beam, or perform fine motor tasks independently with each hand. When attempting unilateral hand movements, mirror movements occur in the opposite hand. There is no history of seizures. Physical examination showed a height of 132.2 cm (above the 97th percentile), a weight of 30.0 kg (above the 97th percentile), a head circumference of 52.0 cm, and sexual development corresponding to Turner stage 2. The LHRH provocation test confirmed the diagnosis of central precocious puberty. Cranial magnetic resonance imaging (MRI) revealed cortical hypertrophy with irregularities, midline shift, dilated lateral ventricles, hypoplasia of the right frontal lobe, widened and deepened cerebellar sulci within the cerebellar hemispheres, a shortened corpus callosum, and hypoplasia of the cerebellar vermis (Figure 1). The MRI of the sellar region showed no abnormal pituitary signal. The video electroencephalogram (EEG) displayed an absence of dominant rhythm in the occipital region during wakefulness, quiet, and closed-eye states, with a mixed low-amplitude activity of 6–8 Hz interspersed with occasional low-amplitude fast waves. There was approximate symmetry between the right and left hemispheres, and no bilateral epileptiform discharges were observed (Figure 2). The fourth edition of the Wechsler Intelligence Test yielded a verbal comprehension index, perceptual reasoning index, working memory index, processing speed index, and total IQ of 96, 76, 73, 74, and 76, respectively.

**Figure 3:**
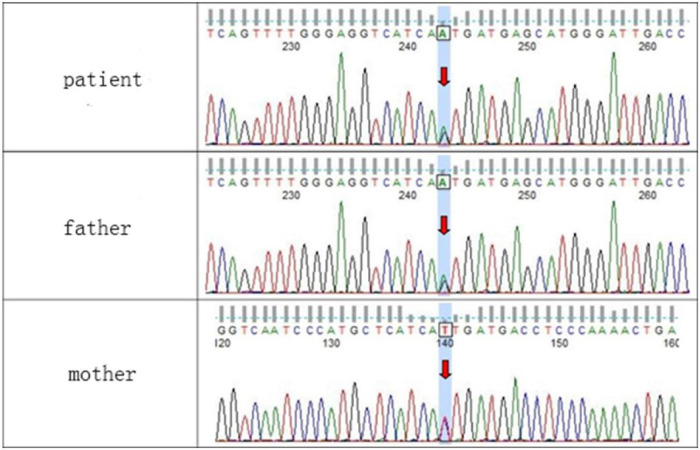
Sequencing validation results for the c.74G > A (p.Ser25Asn) variant in the TUBB2B gene.

**Figure 4:**
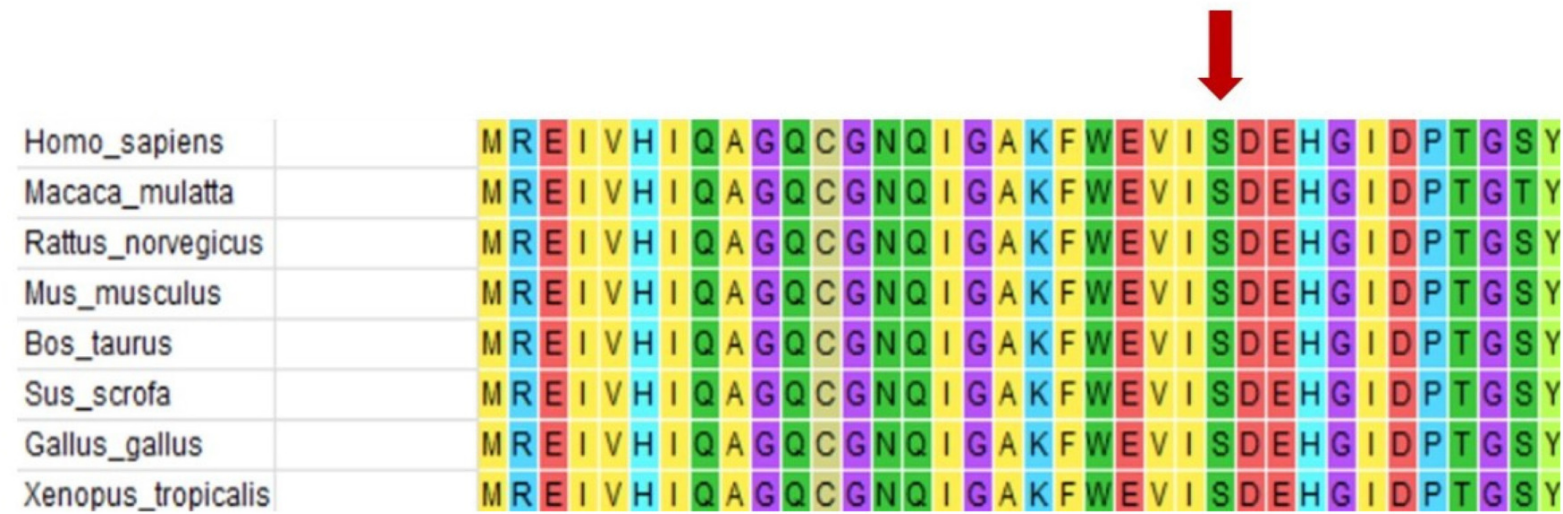
Protein conservation analysis shows that serine 25 of TUBB2B (shown by the red arrow) is a highly conserved amino acid.

**Figure 5:**
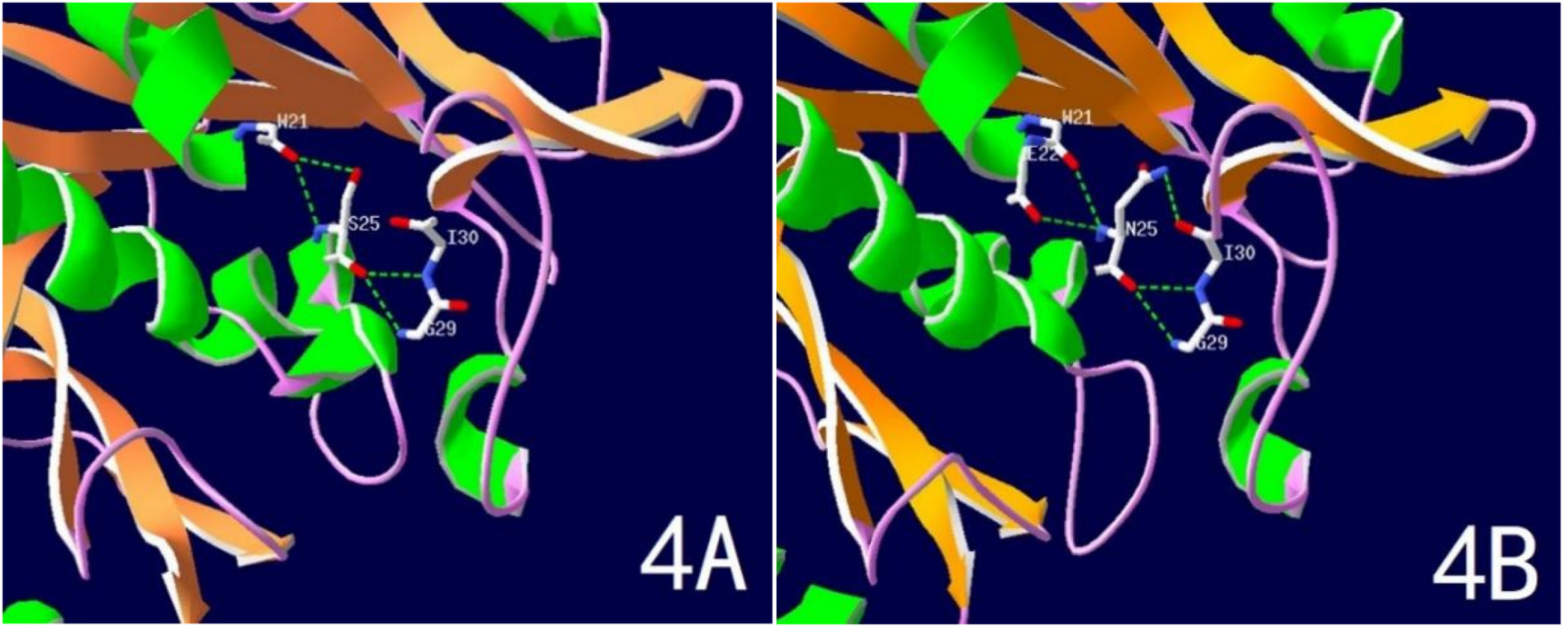
Predicted spatial structure of TUBB2B protein. **(A)** Normal TUBB2B protein structure; **(B)** Ser25Asn variant TUBB2B protein structure.

WES testing revealed that the girl carries a heterozygous variant, c.74G > A (p.Ser25Asn), in the TUBB2B (Tubulin Beta2 B Class IIb) gene. This variant was not detected in either of her phenotypically normal parents (Figure 3). The ACMG criteria and guidelines classified the variant as potentially pathogenic (3). A search through the Human Gene Mutation Database and ClinVar did not identify this specific variant locus. The serine at position 25 of the TUBB2B protein is a highly conserved amino acid across different species, including human, porcine, bovine, mouse, and chicken, as determined by a protein sequence analysis using MEGA-X (Figure 4). Furthermore, protein structure prediction indicated that serine at position 25 of the wild-type TUBB2B protein engages in hydrogen bonding with Trp21, Gly29, and Ile30. The c.74G > A variant disrupts the formation of these hydrogen bonds, which could potentially alter the secondary structure of the protein and thereby impact its function (Figure 5).

Currently, there is no definitive treatment for this condition; management primarily involves addressing the symptoms based on the lesions that manifest.

## 3 Discussion

In this case, although it was accompanied by central precocious puberty, given that the child's predicted final height falls within the normal range and there is no discrepancy when compared to the target height, and considering there are no psychological or behavioral issues directly attributed to the precocious puberty, intervention with GnRHa has not been pursued. It is recommended to engage in regular monitoring of bone age and the rate of height growth.

A mutation rendering microtubulin and microtubule-associated proteins ineffective leads to a tubulinopathy called complex cortical dysplasia. Microtubules, composed of microtubulin and dynamic cytoskeletal polymers, are pivotal in the critical development of cellular processes such as neuronal proliferation, migration, and organization of the cortical layer (4). Neuronal migration is a fundamental aspect of cerebral cortex development. The eukaryotic tubulin superfamily comprises six distinct members—α-, β-, γ-, δ-, ε-, and ζ-tubulin (3, 5, 6), —of which α- and β-tubulin are the most prevalent in humans and form the primary proteins in microtubules. These two types of microtubulin, which constitute approximately 80%–95% of total microtubulins, share similar three-dimensional structures and tightly bond into dimers that serve as subunits for microtubule assembly (7).

Several genes encoding microtubulins are expressed in the developing brain, each governed by specific spatial and temporal patterns. Mutations in seven genes encoding α- (TUBA1A), β- (TUBB2A, TUBB2B, TUBB3, TUBB4A, TUBB5), and γ- (TUBG1) microtubulin are linked to extensive overlapping brain malformations or tubulinopathies (8–10). Bahi-Buisson et al. have comprehensively characterized this wide spectrum, identifying dysmorphic basal ganglia as the most consistent neuroimaging feature (present in 75%–100% of cases depending on cortical malformation subtype) (11). The TUBB2B gene (12), located on chromosome 6p25.2 and spanning four exons, encodes a β-microtubulin composed of 445 amino acids. β-Microtubulin comprises three structural domains: an N-terminal guanine nucleotide-binding region, an intermediate structural domain with one GTP-binding site, and a C-terminal structural domain. Highly expressed in the brain, mutations in the TUBB2B gene can disrupt the normal function of microtubules by interfering with α and β microtubule protein interactions and binding to GTP. Consequently, the mutated TUBB2B protein acts in a dominant-negative manner (OMIM: https://omim.org), impairing the formation of functional α/β microtubule heterodimers and disrupting neuronal migration (13). As demonstrated by Mutch et al., these microtubule disruptions lead to characteristic imaging patterns including: 1. dysmorphic basal nuclei with abnormal anterior commissure orientation, 2. predominant central pachygyria, and 3. frequent cerebellar hypoplasia (14).

Over 40 TUBB2B missense mutations have been identified in the HGMD and ClinVar databases, the majority associated with cortical abnormalities. Literature searches have shown (15–18) that TUBB2B-related disorders predominantly feature: 1. cortical malformations ranging from focal polymicrogyria to widespread tubulin-related dysgyria, 2. dysmorphic basal ganglia with characteristic anterior commissure abnormalities, and 3. variable cerebellar hypoplasia. The cortical phenotype typically shows a gradient of severity, most commonly presenting as asymmetric anterior-predominant polymicrogyria-like dysgyria, central pachygyria with simplified gyral pattern, and complete lissencephaly. The corpus callosum may be affected to varying degrees, from near complete dysplasia to a normal phenotype. Identifiable cerebellar and cerebral abnormalities, such as hypoplasia of the superior cerebellum, particularly in the cerebellar vermis, are the most characteristic features. Most cases exhibit a severe clinical phenotype; however, milder phenotypes have also been reported. In 2022, Chinese scholars described a 27-week-old fetus with the p.L253Q variant of the TUBB2B gene, with fetal MRI revealing agenesis of the corpus callosum, cerebellar hypoplasia, anencephaly, and abnormal cortical development (19).

The patient in this case carries a heterozygous mutation in the TUBB2B gene (c.74G > A, p.Ser25Asn). Ser25 of TUBB2B is a highly conserved amino acid, and structural predictions suggest that the c.74G > A mutation impacts hydrogen bonding formation, leading to alterations in the protein's secondary structure and function. The child exhibits typical CCD imaging findings, abnormal electroencephalogram patterns, and significant intellectual disability. Notably, the child also presents with central precocious puberty, a feature not previously reported in the literature. Brain MRI revealed bilateral thalamic hypertrophy and a nodular shadow in the pineal region, suggestive of a pineal cyst. While the pituitary gland appeared structurally normal, the presence of abnormalities in both the hypothalamus (indicated by thalamic hypertrophy) and pineal region suggests involvement of the sellar region. Given the regulatory roles of the thalamus and pineal gland in the hypothalamic-pituitary-gonadal (HPG) axis, these findings provide a potential neural mechanism for CPP in this case. Lesions in this area are one of the primary causes of central precocious puberty. Nevertheless, further evidence is required to establish a causal relationship between this mutation and central precocious puberty, which is a limitation of this case.

The clinical significance of this case lies in recognizing the complexity of central precocious puberty etiology. After examining the sellar region of the brain in children with central precocious puberty, vigilance must be maintained for other cranial lesions, particularly in cases of early-onset central precocious puberty accompanied by intellectual disability and abnormal motor function. Whole cranial magnetic resonance imaging may be considered in addition to sellar MRI if necessary.

## 4 Conclusion

In conclusion, we have elucidated the genetic etiology of a child with CCD. The aggregation and analysis CCD cases will aid in delineating the clinical phenotypic spectrum and exploring the genotype-phenotype relationship. The potential causal link between TUBB2B gene mutations and central precocious puberty, as well as the specific mechanism of this association, require further corroboration at both pathological and molecular levels.

## Data Availability

The original contributions presented in the study are included in the article/Supplementary Material, further inquiries can be directed to the corresponding authors.
